# Thermal Rectification in Telescopic Nanowires: Impact of Thermal Boundary Resistance

**DOI:** 10.1021/acsami.4c14920

**Published:** 2024-12-18

**Authors:** Yashpreet Kaur, Saeko Tachikawa, Milo Yaro Swinkels, Miquel López-Suárez, Matteo Camponovo, Alicia Ruiz Caridad, Wonjong Kim, Anna Fontcuberta i Morral, Riccardo Rurali, Ilaria Zardo

**Affiliations:** † Department of Physics, Universität Basel, Basel 4056, Switzerland; ‡ Institut de Ciència de Materials de Barcelona, ICMAB-CSIC, Campus UAB, Bellaterra 08193, Spain; § Laboratory of Semiconductor Materials, École Polytechnique Féderale de Lausanne, Lausanne 1015, Vaud, Switzerland; ∥ Swiss Nanoscience Institute, Basel 4056, Switzerland

**Keywords:** thermal rectification, thermal boundary resistance, telescopic nanowire, GaAs, thermal conductivity, thermal bridge

## Abstract

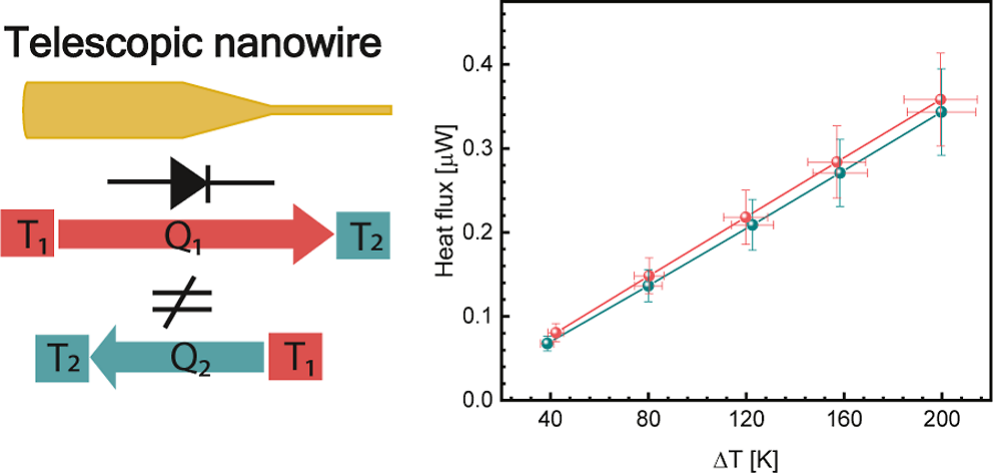

A thermal diode, which, by analogy to its electrical counterpart, rectifies heat current, is the building block for thermal circuits. To realize a thermal diode, we demonstrate thermal rectification in a GaAs telescopic nanowire system using the thermal bridge method. We measured a preferred direction of heat flux, achieving rectification values ranging from 2 to 8% as a function of applied thermal bias. We demonstrate that the thermal boundary resistance between the thin part with the wurtzite crystal phase and the thick part with the zinc-blende crystal phase of the telescopic nanowire plays a crucial role in determining the amount and direction of heat flux rectification. This effect is confirmed by numerical solutions of the one-dimensional heat equation based on ab initio data. Additionally, we accounted for the effect of the thermal contact resistance. This work is the first experimental indication of rectification using a telescopic nanowire where we reveal the importance and role of the thermal boundary resistance in determining thermal rectification.

## Introduction

Photonics and electronics have reached a high level of control and sophistication in technology today, and nanoscale devices have made their way to the industry.^1,2^ Having the same degree of control with phonons, which are the main carriers of heat, will enable us to tackle the problem of overheating frequently arising in densely packed electronic devices. However, heat management, which is a limiting factor to downscaling, performance, and lifetimes of devices, remains challenging.^3,4^ In the past decades, researchers have worked on diverse heat management strategies using thermoelectrics, heat guides, and phononic crystals, to name a few.^5−8^ To achieve reliable control over heat flow, the stepping stone is the realization of an efficient thermal diode, i.e., a device that enables heat to flow preferentially in one direction, thus giving thermal rectification. Furthermore, these devices could be used to avoid overheating of densely packed systems in current microchips with fluctuating heat currents.^9^

Efforts were made as early as the 1930s to study heat rectification for the copper/cuprous oxide interface caused by asymmetric electronic heat transport.^10^ This work was followed by several other theoretical and experimental studies for thermal rectification based on conduction, convection, and radiation.^11,12^ A necessary condition for achieving thermal rectification is breaking the symmetry of the system along the transport direction. Some of the strategies used for rectification include thermal warping/strain,^13−15^ different work functions of the materials,^16^ coupling nonlinear lattices,^17,18^ nonuniform mass loading,^19^ exploiting temperature- and spatial-dependent thermal conductivities,^20−23^ defect engineering,^24^ metal–insulator transition,^25^ and the use of phase change materials.^26−28^ The above-mentioned rectification mechanisms led to rectification factors ranging from 7 up to 50%, with an exceptionally high rectification factor of 96% for MoSe_2_–WSe_2_ heterostructures.^29^ While the rectification factor reported here does not compete with these values, we are reporting the proof of principle and demonstrating the effectiveness of a novel rectification mechanism, which can be further applied to other systems and rectification schemes.

In junctions of two materials, thermal rectification stems from the different nonlinear temperature dependence of the thermal conductivity κ(*T*) of the two materials.^30^ Nanostructuring further helps to tune the thermal conductivity due to phonon scattering effects, resulting in an additional control factor for thermal rectification, as explored in refs (31–33). These two approaches (heterojunction with nanostructuring) are at the core of two theoretical works demonstrating thermal rectification in nanowires (NWs) by molecular dynamics simulations with promising results.^34,35^ Specifically, these theoretical proposals considered telescopic NWs, which are filamentary structures with an abrupt change in diameter.

Here, we exploited the size and temperature dependence of the thermal conductivity to manipulate heat flux and study thermal rectification experimentally. Most importantly, we have leveraged the thermal boundary resistance to tune the direction and size of thermal rectification, as suggested in previous theoretical works.^36^ For this purpose, we investigated the thermal properties of semiconducting gallium arsenide (GaAs) telescopic NWs.^37^ The investigated NWs are 10 to 20 μm long with diameters of approximately 300 and 90 nm in the thick and thin parts, respectively. The thick part of the NW is grown under gallium-rich conditions and exhibits a predominantly zinc blende (ZB) crystal structure. In contrast, the thin part of the NW exhibits a wurtzite (WZ) crystal phase (see the Supporting Information for more details). As a result, the telescopic NWs studied here are made of two segments with different diameters and polytypes. These two features, even taken individually, result in different κ(*T*).^38,39^ To perform accurate thermal transport measurements on these NWs, we chose the so-called thermal bridge method, consisting of suspended silicon nitride (SiN_*x*_) membranes with Joule heaters to apply thermal bias and measure the thermal conductance^40−44^ among many other thermal measurement methods.^45−49^

## Results and Discussion

### Telescopic NWs as a Thermal Rectifier

**Figure 1:**
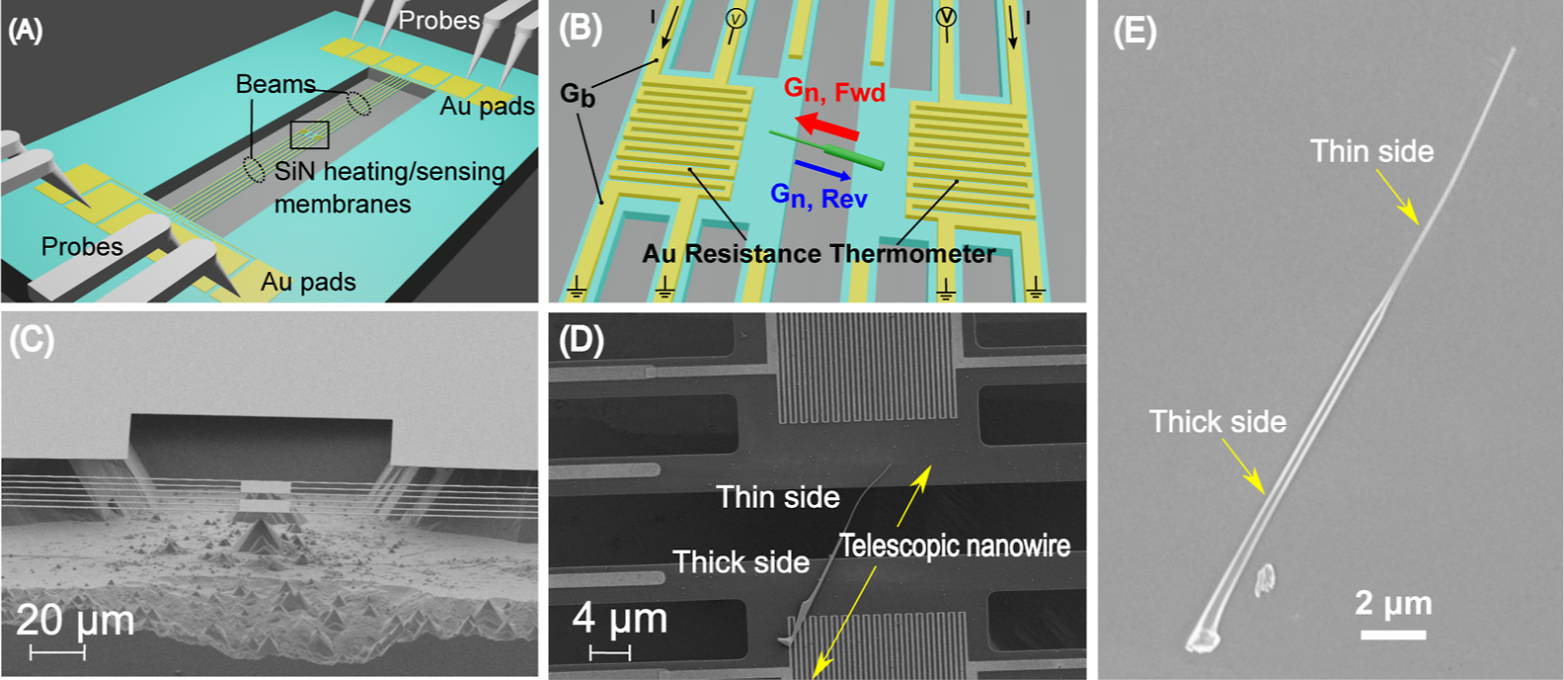
(A) Schematic of a suspended platform device. (B) Zoomed-in schematic of the active part of the device with a NW. (C) SEM image of a suspended thermal bridge device as seen from the diagonal view. (D) Microscopic image giving a top view of a telescopic NW bridged between the suspended platforms for thermal transport measurements. (E) SEM image of a telescopic NW displaying the thick and the thin part with the transition in between.

The thermal properties of the telescopic NWs were studied using thermal bridge devices with gold (Au) coils functioning as microjoule heaters/sensors patterned on SiN_*x*_ membranes with long SiN_*x*_ beams for thermal isolation. A schematic of these devices can be seen in Figure 1A,B. These devices are widely used to study the thermal transport properties of small samples like NWs or thin films by bridging them between the membranes to achieve precise control over the thermal bias and sensitive temperature measurements by employing the linear dependence of the electrical resistance of metal coils with temperature.^40^ A scanning electron microscopy (SEM) image of the fabricated device is shown in Figure 1C. Figure 1D displays one of the investigated telescopic NWs bridging the two suspended membranes. Figure 1E shows the SEM image of a telescopic NW with a thick and a thin part with a tapering transition in between. Further details on the device fabrication and characterization can be found in the Supporting Information.

Measurements were performed in a vacuum environment of the order of 1 × 10^–5^ mbar to avoid any heat transport through convection. The devices were initially calibrated in the temperature range of interest (300 to 360 K). We paid particular attention to examining the asymmetry in the beam conductances of each membrane caused during the fabrication process. The asymmetry of the device was assessed as part of device characterization and taken into account in calculating the thermal conductances in the forward and reverse directions (see the Supporting Information for more details).

The thermal conductance of the NWs was measured by using the standard thermal bridge method. Electric current was applied from the contact pads and sent through the beams to the coils on one of the membranes, causing a rise in the temperature, Δ*T*_H_, by Joule heating with respect to the base temperature. This membrane functions as a heater platform. Due to heat transport through the NW, a slight temperature rise, Δ*T*_S_, can be measured on the second membrane, operated as the sensor platform. The slopes of the temperature rise on the heater and the sensor side as a function of the power dissipated are used to calculate the thermal conductance of the NWs, *G*_n_, given as (detailed physics of the device is discussed in the Supporting Information)
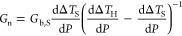
1where *G*_b,S_ is the beam conductance given as
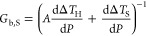
2Here, *A* = *G*_b,Rt_/*G*_b,Lt_ is the asymmetry factor of the device measured before placing the NW as a part of the device characterization (detailed description is in the Supporting Information), and *P* is the total power dissipated in the heater and sensor. Several thermal bridge devices were characterized for their asymmetry, and some are listed in Table S1. Devices showed a range of asymmetries that can be attributed to fabrication process disparities. Among the available devices, we carefully chose devices with the smallest possible asymmetry for thermal rectification measurements (e.g., device 1 in Table S1 with an asymmetry factor (1.026 ± 0.008)). We switched the heater and the sensor sides by reversing the temperature bias and measured *G*_n_ again to estimate thermal rectification. The asymmetry factor (A) is then also taken into account for thermal rectification calculations, as a correction factor to prevent overestimation of the thermal rectification. The sources of measurement error and sensitivity of our measurement setup are discussed in detail in the Supporting Information.

**Figure 2:**
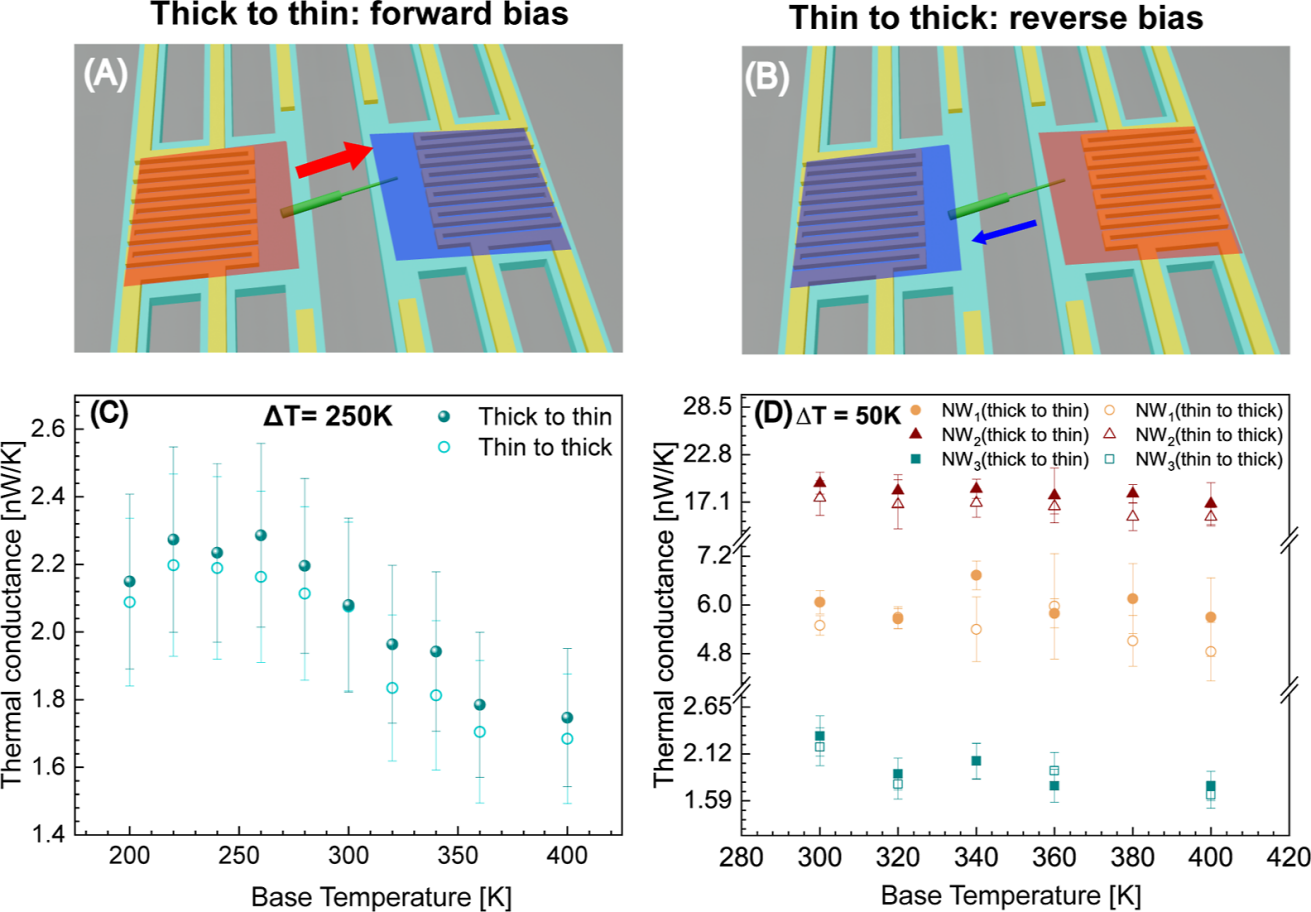
(A,B) Schematic of thick-to-thin (forward bias) and thin-to-thick (reverse bias) configurations of thermal transport measurement. (C) Thermal conductance of a telescopic NW (NW_3_) as a function of base temperature measured with an applied thermal bias of 250 K. (D) Thermal conductance of three different telescopic NWs in the temperature range of 300 to 400 K at an applied thermal bias of 50 K.

To study thermal rectification, we measured the thermal conductance of several telescopic NWs. We applied temperature bias in both directions by flowing heat current from the thick to the thin side of the NW (thick-to-thin) and reverse (thin-to-thick), as shown in Figure 2A,B, and investigated their temperature dependence. The thermal conductance for a telescopic NW in the range 200–400 K, for an applied temperature bias of 250 K, increased from 200 to 220 K, then saturated between 220 and 260 K, and decreased above 260 K, as shown in Figure 2C. The temperature bias was then reversed to get the thermal conductance of the wire in the opposite direction, which followed the same trend. However, the thermal conductance values in most cases were systematically different between the two conditions. Specifically, we found them to be higher along the thick-to-thin than along the thin-to-thick direction. This can also be seen in Figure 2D, showing the same behavior for three different NWs (NW_1,2,3_) for an applied thermal bias of 50 K as a function of base temperatures from 300 to 400 K. It is worth noticing that the measurement conditions were further improved for experiments after measuring NW_1_. Furthermore, NW_3_ exhibits the highest thermal contact resistance, whose contribution is discussed more thoroughly below. This reproducible and systematic observation of the preferential direction of heat flux in several measured NWs where |*G*_n_(Δ*T*)| ≠ |*G*_n_(−Δ*T*)| is a clear signature of rectification.

The observed temperature dependence is consistent with the expected behavior of a material where boundary scattering plays a non-negligible role without being the dominant scattering mechanism. Indeed, we observe that the thermal conductance at first increases with temperature, following the specific heat.^39^ Above a certain temperature, on the other hand, umklapp scattering takes over and the thermal conductance decreases. A similar dependence on temperature is observed also on a 300 nm-thick ZB segment in a temperature range between 270 and 375 K (see the Supporting Information). Although the overall temperature dependence in a telescopic NW is that of a bulk material and not the one of an ultrathin NW, where phonon transport would be limited by temperature-independent boundary scattering,^50^ first, the thermal conductance transition from specific heat dominated to umklapp dominated occurs at a much higher temperature, i.e., 250 K, than what occurs in bulk GaAs; second, the measured thermal conductivity is lower than the values reported for bulk GaAs.^38,51^

**Figure 3:**
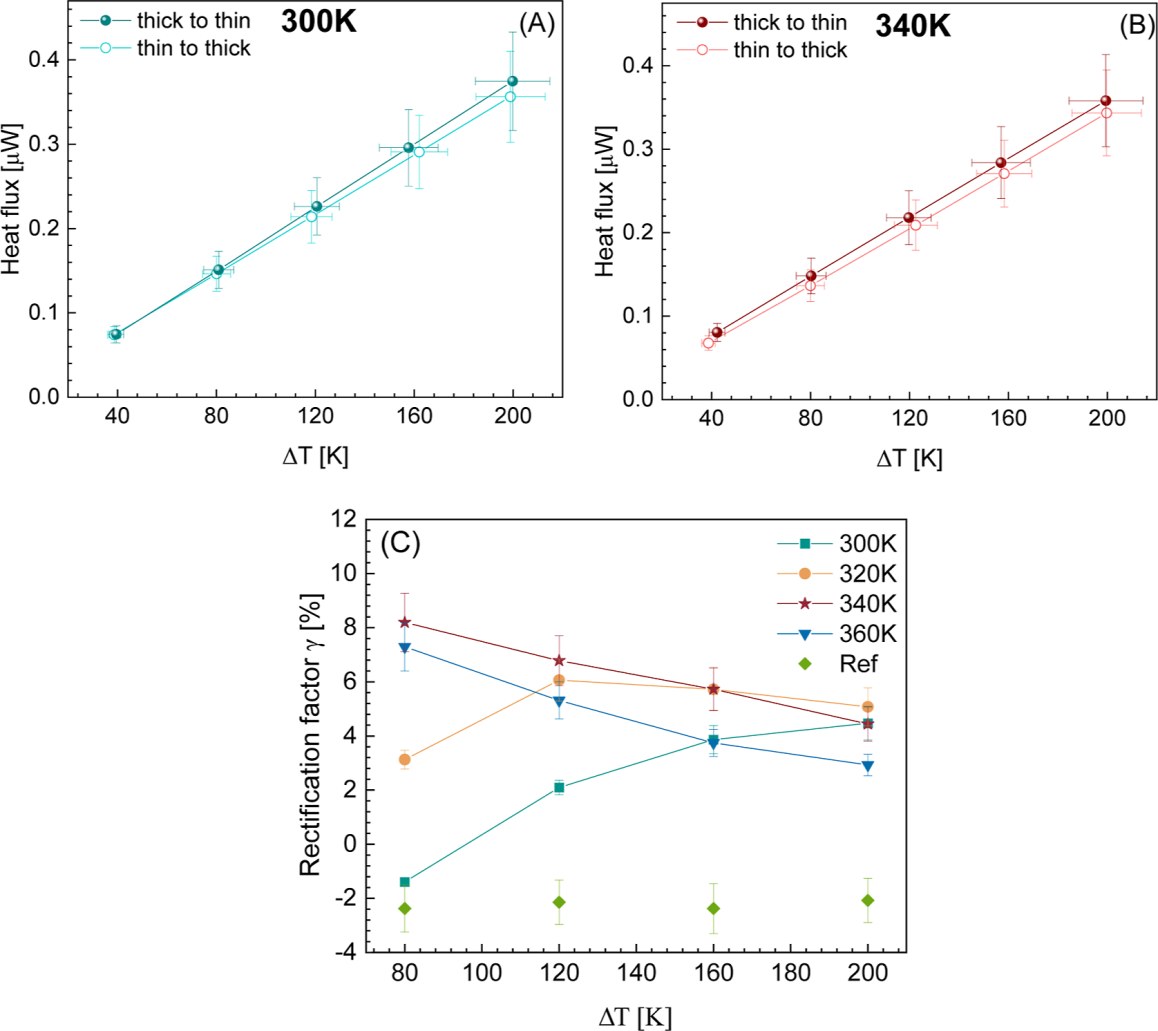
(A,B) Diode curve of a telescopic NW (NW_3_) at base temperatures of 300 and 340 K as a function of applied temperature bias. (C) Measured rectification factor in telescopic NW (NW_3_) as a function of the applied temperature bias for base temperatures ranging from 300 to 360 K.

Figure 3A,B display the diode curves, plotting the heat flux as a function of applied temperature bias at two example base temperatures, 300 and 340 K. The heat flux increases upon increasing the temperature in both directions, but the increase is always higher in the thick-to-thin, i.e., forward direction. This effect becomes stronger with temperature bias. The heat flux is simply calculated as *Q* = *G*_n_Δ*T*, where *Q* is the heat flux, *G*_n_ is the NW’s thermal conductance, and Δ*T* is the measured temperature bias.

In our experiments, we define the direction of heat flux from the thick part to the thin part as the forward direction, as shown in schematic 2A. Consequently, the heat flux in the direction of the thin part to the thick part is defined as the reverse direction, as shown in 2B. Therefore, the thermal rectification factor is defined as
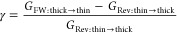
3

A systematic study of rectification measurements for several base temperatures in NW_3_ as a function of temperature bias is displayed in Figure 3C and compared with the reference. The reference is a thermal bridge device before suspending the NW between the membranes. The asymmetry of the device leads to a constant rectification (using the same formulation as defined in eq 3) of approximately −2% as a function of temperature bias. It is worth noticing that this device rectification (green diamond-shaped data points in Figure 3C) is independent of temperature and occurs in the opposite direction than the one of the telescopic NW. The rectification values from the telescopic NW are higher than those from the reference system. This implies that we can measure rectification coming from our sample system of telescopic NWs with this measurement method. From the telescopic NW, the rectification values obtained at an applied bias of up to 120 K are spread between −1.8 and 8%. On the other hand, as the thermal bias increases, the rectification converges to a value between 2.4 and 4.4%. At a 300 K base temperature, the rectification value goes from negative to positive, increasing with temperature bias. This transition from a negative to a positive value at 300 K, as well as the convergence later, is unexpected and nontrivial.

### Theoretical Models and Calculations for Estimating Thermal Rectification

**Figure 4:**
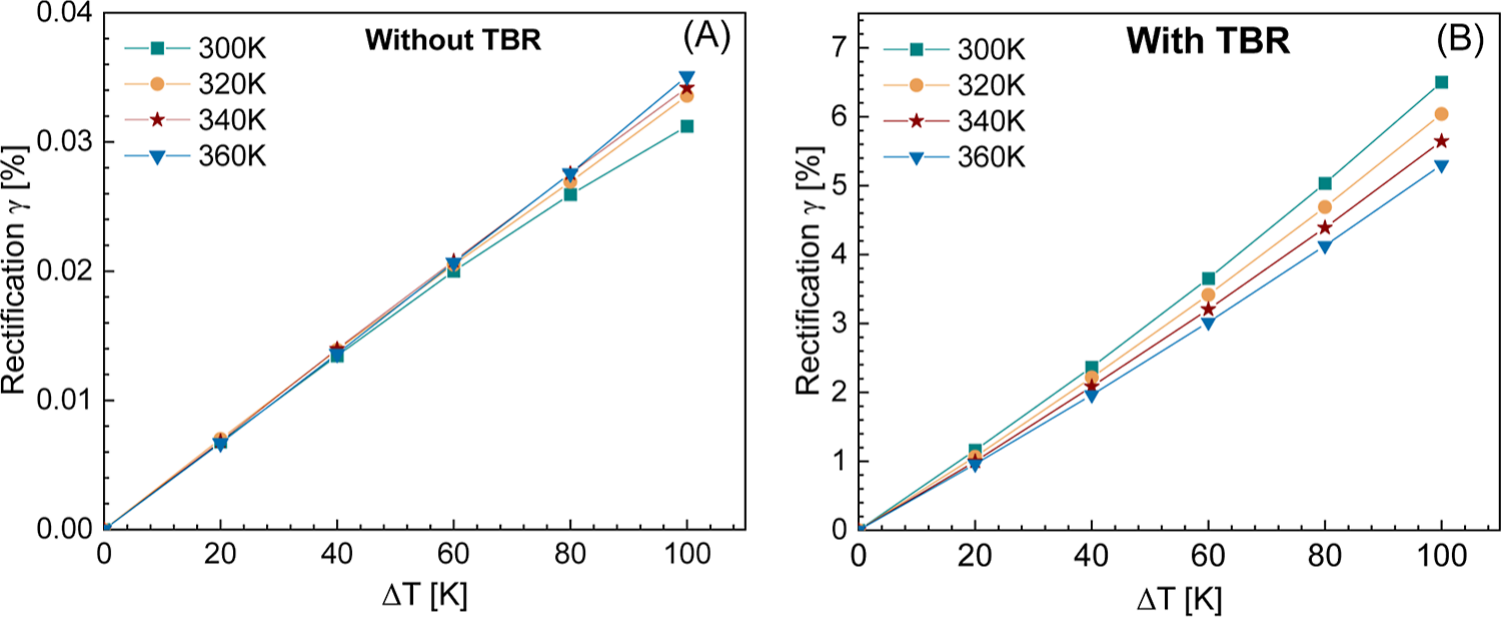
Rectification values as calculated from the theory numerically solving the heat equation with a finite-element method (A) without TBR and (B) with TBR as a function of the applied temperature bias for different base temperatures between 300 and 360 K and for the geometry of the NW_3_ of Figures 2C and 3.

As mentioned above, thermal rectification in heterojunctions is commonly understood in terms of the different temperature dependence of the thermal conductivity, κ(*T*), of the two constituent segments. These systems are well described by the linearized model of Dames,^52^ which shows that if the thermal conductivity of the two materials depends on the temperature through a power law and the interface thermal resistance can be neglected, then the maximum degree of thermal rectification is
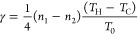
4where *T*_H_, *T*_C_, and *T*_0_ are the hot, cold, and average temperatures, while *n*_1_ and *n*_2_ are the exponents of the κ(*T*^*n*^) of each material. Such a maximum can be achieved when the condition of resistance matching is met, i.e., when the thermal resistance of the two segments is approximately equal. We have fed the model of Dames with κ(*T*) of ZB and WZ GaAs computed ab initio, solving the Boltzmann transport equation beyond the relaxation time approximation and accounting self-consistently for the boundary scattering resulting from the radial confinement imposed by the NW geometry (see ref (38) and the Supporting Information for full details of the calculations). The same computed κ(*T*) is also used as inputs of an in-house finite-element code that solves numerically the heat equation^34^ and allows bypassing some of the simplifying assumptions used by Dames, such as the power-law dependence of the thermal conductivity. The two solutions exhibit good qualitative agreement (see the Supporting Information), suggesting that the approximation used in the model of Dames is sound and does not prevent to capture of the general trend of the rectification. However, they both disagree with the experimental results and predict rectification factors that are 2 orders of magnitude lower (see Figure 4A, where we display the numerical finite-element solution of the heat equation; Figure S6 reports results obtained from the model of Dames,^44^ where, besides neglecting the thermal boundary resistance (TBR), an exponential dependence of the thermal conductivity is assumed).

As a next step, we considered the effect of the interface thermal resistance, also known as TBR,^53,54^ between the two segments, as it was reported that it could sometimes dominate thermal rectification effects in nanoscale systems.^36^ In particular, this can happen when the resistance-matching conditions, assumed in the derivation of eq 4, are not met,^41^ as we expect to be here. In such a case, the rectification also stems from the temperature dependence of the TBR and from the fact that the temperature of the interface, *T*_i_, changes when the thermal bias is reversed (while when the resistance-matching condition holds, *T*_i_ = (*T*_H_ + *T*_C_)/2, and this effect vanishes). We proceeded as follows: At first, we calculated the TBR of a bulk ZB-GaAs/WZ-GaAs junction within the diffusive mismatch model^53^ and used our ab initio data for the phonon frequencies and velocities. Next, we included the DMM-TBR term in our in-house finite-element code and solved self-consistently the heat equation. Within this more general approach, we obtained thermal rectifications of the same order as those observed experimentally, as shown in Figure 4B.

These results suggest that a standard model, where the thermal rectification exclusively depends on the different temperature dependence of the thermal conductivity of the two segments, cannot account for the experimental results and does not even capture the correct order of magnitude. A fair agreement, recovering the correct order of magnitude, is obtained when considering the TBR. In the Supporting Information, we present an exploration of the segments’ radius–diameter phase space, which shows that if physically sound κ(*T*) is assumed (and not necessarily only those directly resulting from our ab initio calculations), we found that the order of magnitude of the measured thermal rectification can only be recovered when the effect of the TBR is considered. This vouches for the generality of our conclusions. We also observe that our positive direction of rectification at higher gradients is of the same order as the results of Molaei et al.,^35^ who performed molecular dynamics simulation on telescopic and polytelescopic NWs.

These findings further suggest that the experimental results presented in Figure 3C originate from the interplay between two contributions. One is the temperature-dependent thermal conductance of the two segments, and the second is the temperature dependence of the TBR. The direction of rectification is determined by which of these two factors is dominating. We speculate that at smaller biases, the temperature dependence of thermal conductance might be dominant together with the contribution of the contact resistance (as further explained below), resulting in a negative rectification. Upon increasing the temperature bias, the TBR becomes the dominant mechanism. For base temperatures of 340 and 360 K, the decreasing trend of the rectification factor is unexpected as rectification is expected to increase with temperature bias. We speculate that this might be due to some nonlinearities arising while applying subtle biases that could be overshadowed at elevated base temperatures. This effect is possibly mitigated at higher temperature bias, where we reach convergent values of rectification with TBR still being the dominant mechanism.

### Effect of Thermal Contact Resistance

We should address another factor that might play a significant role in thermal conductance and rectification measurements: the thermal contact resistance. It is still a challenge in the field of thermal transport to eliminate the thermal contact resistance, which might lead to an underestimation of the thermal conductance or unexpected effects when measuring thermal properties. To understand the role of thermal contact resistance in our measurements, we performed combined electro-optothermal measurements on the telescopic NWs placed on the thermal bridge device. We probed the local temperature rise on the NW using Raman thermometry at the contacts between the NW and the SiN_*x*_ platforms while electrically applying a temperature bias. These measurements provide us with an estimation of the temperature jump at the contacts at the two NW’s ends. The temperature at the two contacts is measured for both directions of applied temperature bias for each bias condition. The thermal equivalent circuit for a suspended telescopic NW system on a thermal bridge device that we considered for our calculations is illustrated in the Supporting Information.

**Figure 5:**
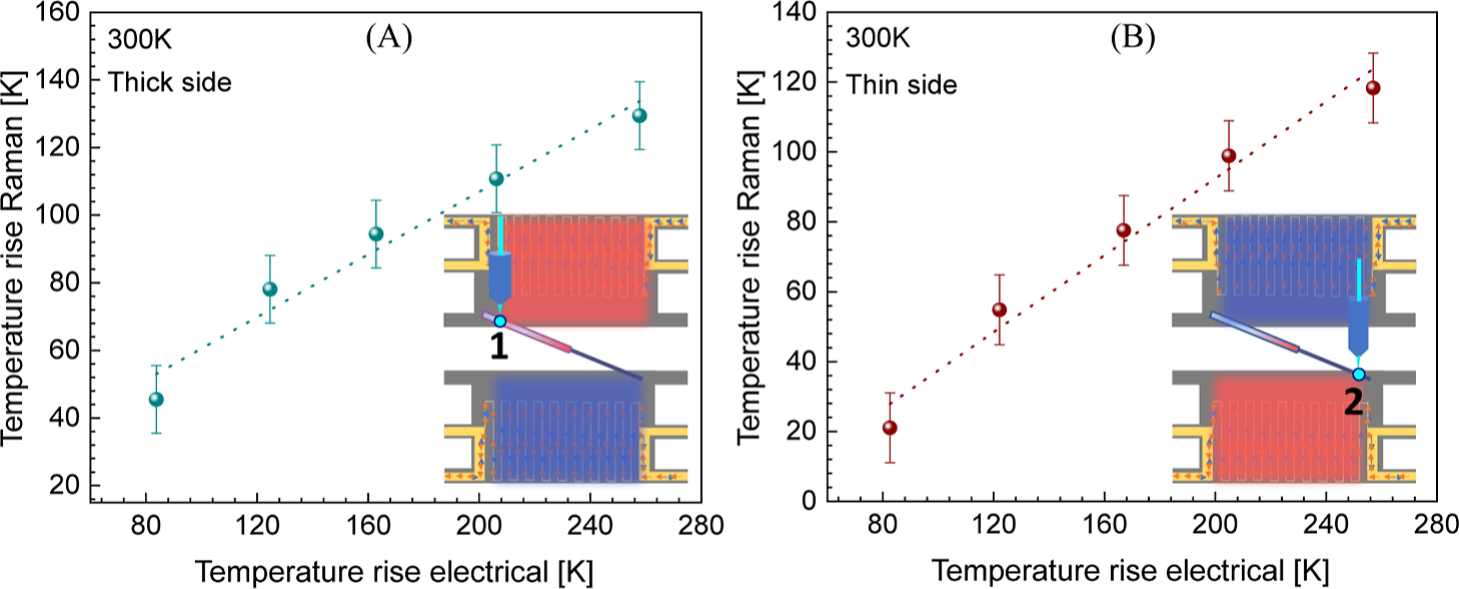
Temperature rise on NW_3_ measured by Raman thermometry at contact points 1 and 2 as a function of electrically applied temperature bias when the high temperature is applied at the contact on the thick (A) and thin (B) side of the NW.

Figure 5 shows the dependence of temperature rise at the contact points (marked as 1 and 2) on the thick (panel A) and the thin (panel B) sides as measured by Raman thermometry as a function of the electrically applied temperature bias. The temperature increases linearly with the electrically applied one, but there is a temperature drop at the contacts on the thick and thin sides. This implies the presence of contact resistance, which reduces the effective applied temperature bias. These measurements, together with a thermal equivalent circuit of our system, enable us to estimate the thermal contact resistance (see the Supporting Information for the thermal equivalent circuit). On the thick side, we measure a contact resistance of (2.88 ± 9.63) × 10^8^ K/W, while on the thin side, we measure a contact resistance of (2.51 ± 7.83) × 10^8^ K/W for the highest temperature at the contacts (i.e., upon application of the highest temperature bias–nominal bias 200 K) at a base temperature of 300 K. However, if we consider the error bars, the contact resistance values are comparable and cannot be differentiated. The large error bars on contact resistance result from the low spatial (900 nm) and temperature resolution (∼10 K) of the Raman thermometry compared to the thermal bridge method. It should be noted that the contact resistance does not induce any nonlinear effects in the thermal conductance measurements at different temperatures and exhibits a rather flat profile except at lower biases (see Figure S8). Therefore, considering the temperature-independent behavior of thermal contact resistance, we can safely exclude that the observed thermal rectification in our case originated from thermal contact resistance between the SiN_*x*_ membranes and NWs except at low biases (300 K at the nominal bias of 80 K (effective thermal bias of 24 K)).

**Figure 6:**
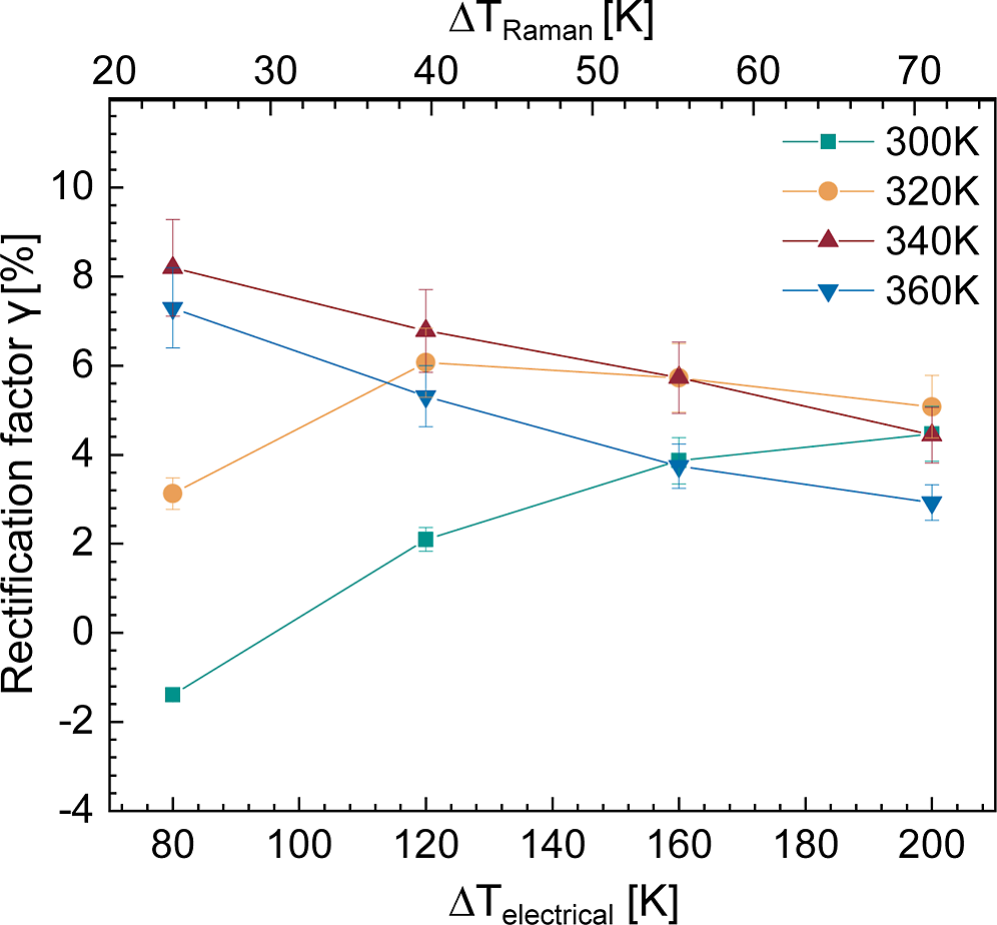
Plot of observed rectification in a telescopic NW as a function of corrected temperature bias measured by Raman thermometry (top axis) and electrically measured temperature bias (bottom axis) for base temperatures between 300 and 360 K.

Since our measurements do not expose any significant nonlinear effects due to the contact resistance, we conclude that it does not significantly contribute to rectification in our case. Therefore, employing the combined electro-optothermal measurement scheme gave us good insight into the local thermal transport in the NW, proving a powerful tool to study contact resistance. Finally, we corrected the offset in the *x*-axis of the electrically applied temperature bias of Figure 3C according to the effective applied temperature bias felt by the NW measured by Raman thermometry, as shown in Figure 6.

In conclusion, we have experimentally demonstrated thermal rectification in a telescopic NW dominated by thermal boundary resistance. In telescopic NW, we measured the preferred direction of heat flow from the thick part to the thin part of the NW, resulting in a maximum thermal rectification up to 8%. The asymmetric heat transport is accounted for by considering the temperature dependence of the thermal conductivity of the two segments and that of the thermal boundary resistance between the thick part (ZB) and the thin part (WZ) of the NW. The latter plays a decisive role in driving thermal rectification. This is supported by theoretical modeling based on ab initio calculations and a numerical solution of the one-dimensional heat equation. The mechanism of thermal rectification in our case is different from the works published before, and it is reported experimentally. This thermal rectification mechanism can be used in other material systems as well, provided the resistance mismatch conditions leading to a temperature-dependent TBR are met. This mechanism can be exploited in combination with other mechanisms to realize higher thermal rectification ratios in novel material systems. The contribution of thermal contact resistance at the contacts between the membrane and the NW was quantified by developing a thermal equivalent circuit model with the unique electro-optothermal measurement setup combining Raman thermometry with the thermal bridge method.

Our findings will impact the future development of thermal circuit elements. The understanding of these underlying physical mechanisms is a significant step forward in the design of elements that can be integrated into low-power electronics for efficient cooling and energy harvesting to power them.

## Experimental Methods

### NW Growth

The III–V GaAs telescopic NWs used for this study were grown using a self-assisted vapor–liquid–solid (VLS) growth mechanism by molecular beam epitaxy technique on periodically patterned arrays on SiO_*x*_/Si(111) substrates.^37^ In order to engineer the abrupt change in diameter, the size of the gallium droplet used to assist NW growth is swiftly decreased by increasing the arsenic flux. This leads to the shrinking of the droplet size, with a consequent change in diameter size from thicker to thinner. See the Supporting Information for more details.

### Fabrication

The thermal bridge devices were fabricated by using laser and electron beam lithography techniques to pattern Au lines and coils. To suspend the devices for thermal isolation, dry and wet etching methods were used to remove SiN_*x*_ and Si underneath, respectively (Supporting Information). Individual NWs were placed between the SiN_*x*_ membranes by micromanipulation.

### Measurement Setup and Details

The electrothermal transport measurements were performed in the ST-500 Janis probe station, which can be operated between 10 and 400 K. The multiprobe tips with six fingers and finger spacing of 100 μm were used to establish electrical contact with the suspended device. Thermal biases were applied by Joule heating the coils on one side of the device using DC current ranging from 1 to 60 μ A using a Keithley 2000 current source, and the voltage drop was measured using Keithley Multimeter 4200. On the sensing side, to measure the temperature rise as a result of heat flowing through the NW, a small AC current of 0.1 μA was sent, and the resistance change was measured using a Zurich instruments lock-in amplifier. This resistance rise is then related to the temperature rise using calibration of the device (see the Supporting Information).

The electro-optothermal measurements of Raman thermometry were performed in the same ST-500 Janis probe station embedded in an optical path. A continuous 488 nm Cobolt Sapphire laser was used as the light source. The laser spot was focused onto the NW with a 100X Mitutoyo (N.A = 0.7) objective lens, resulting in a laser spot of approximately 900 nm. The weak backscattered Raman signal from the NW was guided using mirrors and a collection lens to the single-stage spectrometer consisting of 1800 g/mm grating, which disperses the light onto the CCD. The highly intense elastically scattered Rayleigh signal was filtered using an edge filter before entering the spectrometer. Since the Raman signal was weak, the spectrum was acquired three times at the same spot for an acquisition time of 300 s and observed through the spectrometer software VistaControl. The Raman peak was fitted with a Lorentzian to extract the peak position in cm^–1^.

## Supporting Information Available

Experimental details of NW growth, device fabrication, and characterization; details of theoretical calculations; and thermal equivalent circuit of the telescopic NW (PDF)
