# Supplementary material for: Thermal Rectification in Telescopic Nanowires: Impact of Thermal Boundary Resistance
Source: ACS Appl Mater Interfaces. 2024 Dec 18;17(1):1883–91. doi: 10.1021/acsami.4c14920 (PMC11783544; doi:10.1021/acsami.4c14920)
Supplement: Supplementary file 1 — am4c14920_si_001.pdf [file am4c14920_si_001.pdf]

Supporting Information for

Thermal rectification in telescopic nanowires: impact of thermal boundary resistance

Yashpreet Kaur,[†] Saeko Tachikawa,[†] Milo Yaro Swinkels,[†] Miquel López-Suárez,[‡] Matteo Camponovo,[†] Alicia Ruiz Caridad,[†] Wonjong Kim,[¶] Anna Fontcuberta i Morral,[¶] Riccardo Rurali,[‡] and Ilaria Zardo[*,†,§]

†*Department of Physics, Universität Basel, Basel, 4056, Switzerland*

‡*Institut de Ciència de Materials de Barcelona, ICMAB-CSIC, Campus UAB, Bellaterra, 08193, Spain*

¶*Laboratory of Semiconductor Materials, École Polytechnique Féderale de Lausanne, Lausanne, 1015, Vaud*

§*Swiss Nanoscience Institute, Basel, 4056, Switzerland*

E-mail: ilaria.zardo@unibas.ch

Growth of Telescopic Nanowires

Telescopic nanowires were obtained by self-assisted growth where gallium droplets were used as the catalyst through molecular beam epitaxy in EPFL Lausanne, Switzerland. To obtain spatially homogeneous nanowires in a highly reproducible manner, arrays of holes are defined by electron beam lithography (EBL) using thermal oxide as a mask on Si (111) substrates. Thanks to the nature of the self-catalyzed growth, the NW diameter could be tuned in the middle of the growth by simply modifying the gallium (Ga) and the arsenic As_4 flux. The

Ga droplet, as the NW growth front, plays an essential role in determining not only NW diameter but also crystal phase depending on the contact angles. To transition into the thin nanoneedle part of the NW, the beam equivalent pressure (BEP) of the As_4 flux was abruptly increased while keeping the Ga flux constant. This was done to diminish the size of the Ga droplet instead of entirely consuming it and, thus, reducing the diameter of the NW. After a few minutes, the NWs taper to form a cone, which grows into thin nanoneedles with a uniform diameter while decreasing the As_4 flux. This is how telescopic NWs are obtained. A schematic of telescopic NW growth and an SEM image of the forest of telescopic NWs are displayed in Figs. S1A and B, respectively. Please refer to reference[1] for more details regarding the process conditions and the details of the telescopic NWs. High-resolution transmission electron microscopy (HR-TEM) images and selected-area electron diffraction (SAED) of the telescopic nanowires were performed in a Jeol JEM F200 cFEG TEM/STEM at 200 KeV to determine its crystalline structure. Fig. S1C depicts the composition of 4 HR-TEM images along the telescopic NW, showing the different crystal structures in thick and thin parts. We performed a SAED of the thick and thin parts of the nanowire area inside the dotted circle and we obtained a diffraction pattern corresponding to the zinc blende and wurtzite structures, respectively. The transition region has ZB twinnings and alternating WZ/ZB crystal structures.

Thermal bridge fabrication and characterization

Fabrication

The devices were fabricated on a silicon $< 100 >$ polished substrate. At first, an insulating layer of stress-free silicon nitride SiN_x with a thickness of 590 nm was deposited on the silicon using plasma-enhanced chemical vapour deposition (PECVD). Then, microstructures, specifically contact pads and lines, are patterned using laser lithography followed by metal evaporation and lift-off. Likewise, second lithography patterned 100 nm wide coils with electron beam lithography while aligning them with previous structures. This is again followed

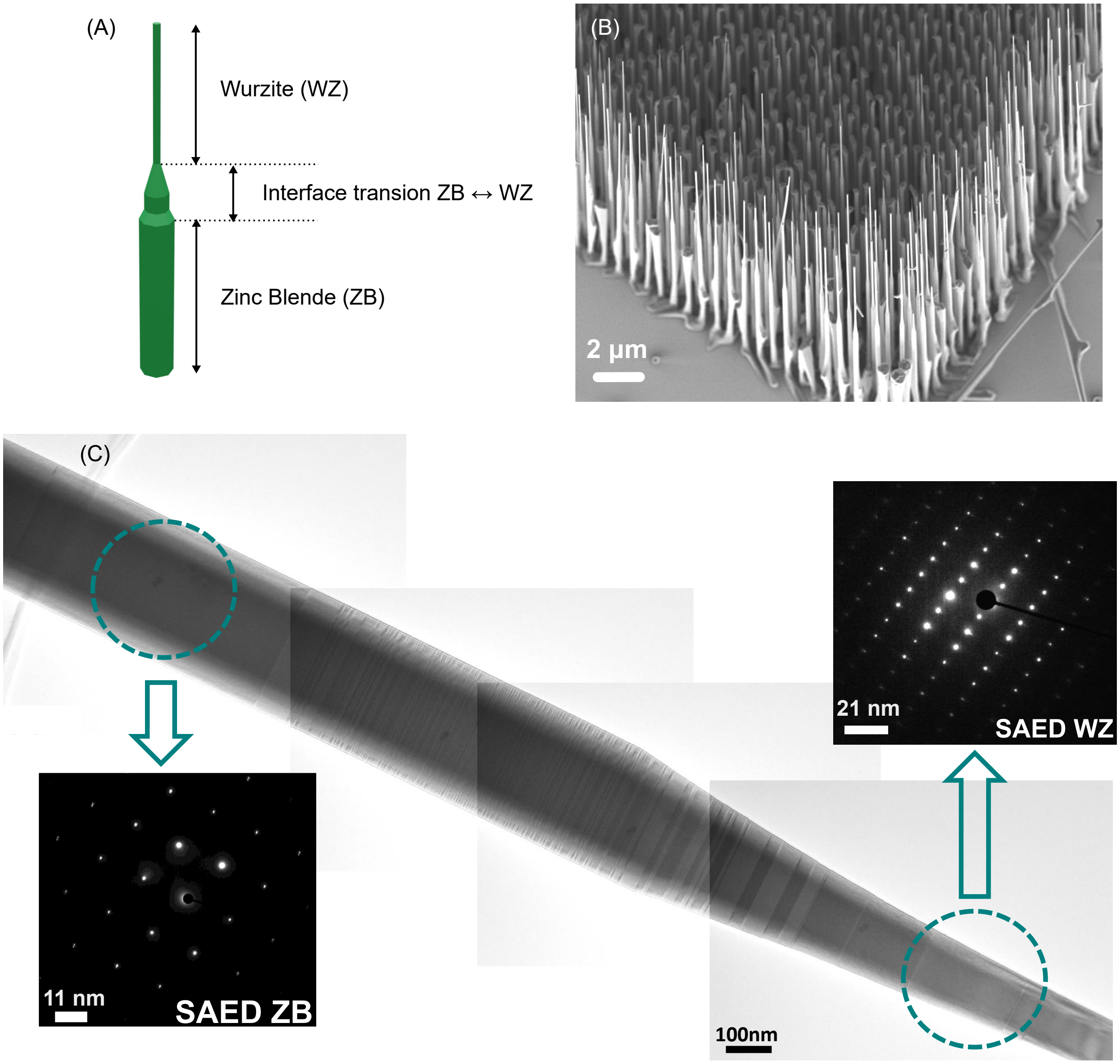

Figure S1. [A] Schematic of a telescopic nanowire. [B] SEM image of a forest of telescopic nanowires with a pitch size of 800 nm. [C] The HR-TEM image of the telescopic NW shows the different crystal structures in thick and thin parts. The transition region has ZB twinnings and alternating WZ/ZB crystal structures.

by metal evaporation and a lift-off step. We used titanium/gold (Ti/Au) with a thickness of 3/27 nm as the metal layer for fabricating our devices. The thin Ti layer was used for good adhesion of Au for the metal structures. After obtaining the full metal structures, the devices need to be suspended by etching. This is carried out by doing another lithography

step in the laser writer to make an opening for the etching step. Further, SiN_x was etched around the gold structures using reactive ion etching with a mixture of trifluoromethane CHF_3 and oxygen at an etching rate of 35 nm/min in the vertical direction. After making SiN_x cut, silicon is exposed in parts to be etched. This is followed by a wet etching step using 2.38 % tetramethyl ammonium hydroxide (TMAH) to isotropically etch silicon at an etching rate of 2 μm/hour at 80 ℃. Therefore, after carrying out this step for 6 hours, the devices were suspended. Since TMAH etches slowly at lower concentrations, the silicon under the platforms is not completely etched. To remove these silicon mountains, a potassium hydroxide (KOH) wet etch is performed for 10 minutes. KOH solution of 33 % by mass in water etches anisotropically in [100] direction at a rate of 1.4 μm/min, creating an undercut. The chips are then rinsed in water to stop the etching and followed by Isopropyl alcohol (IPA) drying to avoid breaking the suspended lines due to capillary action. In the end, we could batch-fabricate up to 400 devices on a chip with a yield of more than 50 %.

Thermal conductance measurements of nanowires using thermal bridge method

To measure the thermal conductance of the nanowire, a high current 1 to 60 μA is sent through the beams to the coils on the heater side, raising the temperature of the heater platform above base temperature ΔT_{H} by joule heating. Due to heat transport through the nanowire, a small temperature rise was measured on the sensor platform ΔT_{S}. A schematic of the parts of the thermal bridge and the respective heat flows are indicated in the main manuscript, Fig. 1A and B. To begin with, the amount of heat flowing through the nanowire is estimated by measuring the power dissipated in the coils. For this purpose, a 4-point measurement is performed. Additionally, a two-point measurement is also conducted to determine the contribution of heat dissipated in the connecting gold lines on SiN_x beams, which partly contributes to the heating of the coils (assuming a linear temperature profile) and, hence, the nanowire. Given that the power dissipated is proportional to the resistance, we can employ the following calculation to estimate the overall power heating the platforms:[2]

$$
\begin{aligned}
P_{\text{total}} &= P_{\text{coils}} + 0.5 P_{\text{beams}} \\
&= \left(1 + 0.5 \frac{R_{\text{beams}}}{R_{\text{coils}}}\right) P_{\text{coils}} \\
&= \left(1 + 0.5 \frac{R_{\text{2point}} - R_{\text{4point}}}{R_{\text{4point}}}\right) P_{\text{coils}}
\end{aligned}
\tag{S1}
$$

Once the total power heating up the platforms is known, we can consider energy conservation for the whole system to estimate the beam conductances as follows:

$$
P_{\text{H}} + P_{\text{S}} = G_{\text{b, H}} \Delta T_{\text{H}} + G_{\text{b, S}} \Delta T_{\text{S}} \Rightarrow G_{b,S} = \frac{P_{\text{H}} + P_{\text{S}}}{\frac{G_{\text{b, H}}}{G_{\text{b, S}}} \Delta T_{\text{H}} + \Delta T_{\text{S}}}
\tag{S2}
$$

where P_{H} and P_{S} are power dissipated in heater and sensor coils; $G_{\text{b, H}}$ and $G_{\text{b, S}}$ are the beam conductances on heater and sensor side; ΔT_{H} and ΔT_{S} are temperature rise on the heater and sensor side with respect to base/ environment temperature, respectively.

We can write another energy conservation for the system composing the nanowire and the sensing side to estimate the nanowire conductance, G_{n} as:

$$
G_{\text{b, S}} \Delta T_{\text{S}} = G_{\text{n}} \left(\Delta T_{\text{H}} - \Delta T_{\text{S}}\right) \Rightarrow G_{\text{n}} = G_{\text{b, S}} \frac{\Delta T_{\text{S}}}{\Delta T_{\text{H}} - \Delta T_{\text{S}}}
\tag{S3}
$$

To obtain conductances, slopes of the temperature rise on the heater and the sensor side are determined as a function of power dissipated. To do so, power is continuously increased in the coils by ramping the current from 1 to 60 μA, causing joule heating to apply several thermal biases. Therefore, assuming a linear relationship between power and temperature, we can write:

$$
\frac{\mathrm{d}\Delta T_{\text{x}}}{\mathrm{d}P} = \frac{\Delta T_{\text{x}}}{P_{\text{H}} + P_{\text{S}}}
\tag{S4}
$$

Substituting Eq. S4 in Eq. S2 and Eq. S3, we obtain $G_{\mathrm{b,\,S}}$:

$$G_{\mathrm{b,\,S}} = \left(A\frac{\mathrm{d}\Delta T_{\mathrm{H}}}{\mathrm{d}P} + \frac{\mathrm{d}\Delta T_{\mathrm{S}}}{\mathrm{d}P} \right)^{-1} \tag{S5}$$

and G_{n} :

$$G_{\mathrm{n}} = G_{\mathrm{b,\,S}}\frac{\mathrm{d}\Delta T_{\mathrm{S}}}{\mathrm{d}P} \left(\frac{\mathrm{d}\Delta T_{\mathrm{H}}}{\mathrm{d}P} - \frac{\mathrm{d}\Delta T_{\mathrm{S}}}{\mathrm{d}P} \right)^{-1} \tag{S6}$$

where $\frac{\mathrm{d}\Delta T_{\mathrm{H}}}{\mathrm{d}P}$ and $\frac{\mathrm{d}\Delta T_{\mathrm{S}}}{\mathrm{d}P}$ are the slopes of temperature rise on the heater and sensor side as a function of total power dissipated. $A = \frac{G_{\mathrm{b,\,Rt}}}{G_{\mathrm{b,\,Lt}}}$ is the asymmetry factor of the device calculated as shown in the section below. G_{n} is the measured thermal conductance of the nanowire.

Characterization

After fabrication, the devices are tested and calibrated for their sensitivity. Further, an assessment of their symmetry is done to evaluate any deviations coming from the fabrication.

Device Calibration: For calibration, the sample with the suspended platform devices is loaded into the Janis probe station. The multiprobe tungsten tips are used to make contact with the devices. A four-point measurement scheme is established to measure the resistance of the coils only while excluding the resistance of the lines and the pads. For the resistance calibration measurement, the temperature of the sample is periodically raised by heating the stage using a temperature controller. A lock-in amplifier MFLI from Zurich instruments reads the resistance while sending a small AC current of 100 nA (to avoid heating by higher currents) at a frequency of 77 Hz.

For metals, the resistance is linearly dependent on temperature above a certain temperature. At low temperatures, as the impurity scattering takes over, it changes the temperature dependence. This might vary between different deposition conditions and the metal purity. The resistance curve for one measured device using a small current (100 nA) is shown in Fig S2 for the left and right bridge in the range 300 to 400 K. The resistance increases linearly

with temperature in this range, as obtained in the measurement. Therefore, the temperature can be mapped in response to resistance change in this temperature range and is used for measurements in this work. This is done for all devices used for measurements of thermal rectification presented in this manuscript. The statistical variance of the calibration of four thermal bridge devices is presented in Fig S2(B), showing the dR/dT values for the left and right bridges for each suspended device. The calibration values range from 2 to 5 Ω/K. This variation in calibration values arises from the fabrication process, leading to some disparities in the devices, which is beyond our control.

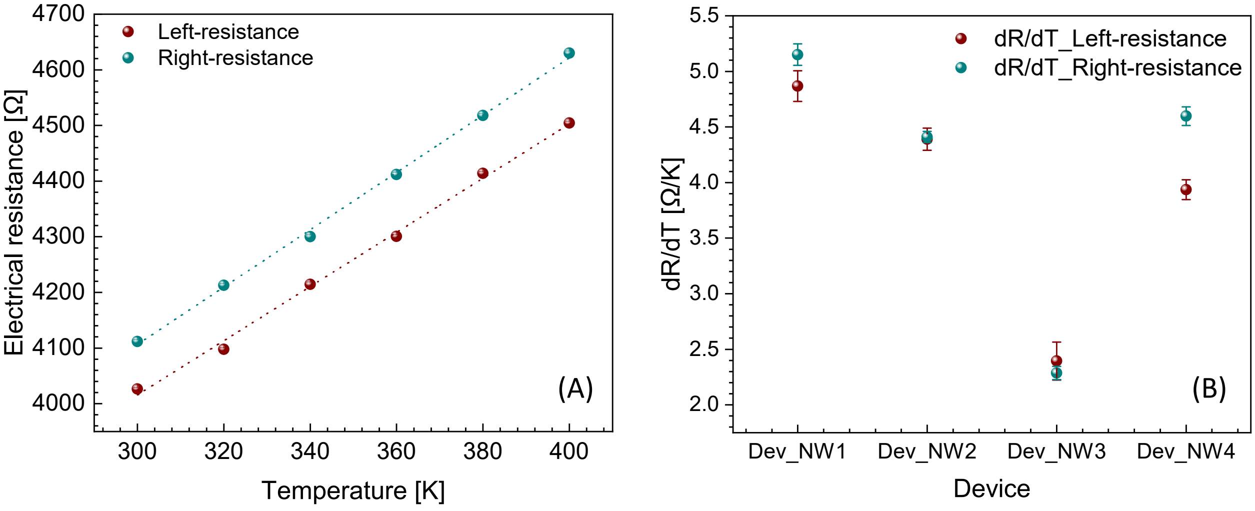

Figure S2. (A) Resistance versus temperature for one of the thermal bridge devices used for rectification measurements in the temperature range 300-400K. (B) Statistics of the dR/dT values of different thermal bridge devices used for the reported measurements in the range 300 to 400 K.

Asymmetry measurement of the Device: To do rectification measurements, we have to ensure that the measured rectification is coming from the sample and not from any other asymmetry in the system, including the device. For this, we analyzed the devices used for the rectification study to account for any asymmetry. This can arise due to limitations or other sources of errors arising during fabrication, leading to unequal conductances of the beams.

Therefore, we write the energy balance equations for the device

$$P_{\mathrm{H}} + P_{\mathrm{S}} = G_{\mathrm{b,\ H}}\Delta T_{\mathrm{H}} + G_{\mathrm{b,\ S}}\Delta T_{\mathrm{S}} \tag{S7}$$

where P_{i} and $G_{\mathrm{b,\ i}}$ are power dissipated and the beam conductance in the heater and the sensor side, respectively, upon applying a temperature bias ΔT. The power is increased continuously on the heater side by increasing the current from 1 to 35 μA, and the slope of temperature with power is determined. If we have an empty device without any sample bridging the two platforms, there will be negligible conduction from one side of the suspended platform to the other. There, the temperature rise on the sensor side is negligible and can be ignored. If we assume a linear relationship between power and temperature, then we can rewrite equation S9 to calculate $G_{\mathrm{b,\ i}}$ for the heating side as

$$G_{\mathrm{b,\ i}} = \frac{dP_{\mathrm{i}}}{d\Delta T_{\mathrm{i}}} \tag{S8}$$

This is performed for both left and right bridges by ramping currents, and $G_{\mathrm{b,\ i}}$ is determined. To calculate the asymmetry, a ratio between the two estimated $G_{\mathrm{b,\ i}}$ values is calculated. A list of estimated $G_{\mathrm{b,\ i}}$ values with respective asymmetry factors is listed below, where i represents the left (Lt) and right (Rt) bridge of the device, respectively.

Table S1. Table of measured asymmetry in suspended thermal bridge devices.

**Device**	$\mathbf{G_{b,Lt}}$**[W/K]**	$\mathbf{G_{b,Rt}}$**[W/K]**	**Asymmetry factor**
1	$(5.57 \pm 0.23)\text{x}10^8$	$(5.72 \pm 0.0.26)\text{x}10^8$	1.026 ± 0.008
2	$(5.53 \pm 0.25)\text{x}10^8$	$(5.78 \pm 0.15)\text{x}10^8$	1.046 ± 0.037
3	$(6.10 \pm 0.52)\text{x}10^8$	$(5.92 \pm 0.48)\text{x}10^8$	0.971 ± 0.012
4	$(9.07 \pm 0.53)\text{x}10^8$	$(9.91 \pm 0.55)\text{x}10^8$	1.092 ± 0.011
5	$(4.88 \pm 0.31)\text{x}10^8$	$(4.70 \pm 0.27)\text{x}10^8$	0.962 ± 0.013
6	$(5.45 \pm 0.31)\text{x}10^8$	$(5.41 \pm 0.24)\text{x}10^8$	0.992 ± 0.009

Sources of measurement error

Several systematic sources of error contribute to the final error of the thermal conductance measurements shown in the main manuscript. This is discussed in detail here.

Thermal Radiation: The effect of thermal radiation between the hot and cold membranes can be determined using the Stefan's law of thermal radiation which is given as:

$$G_{H->Srad} = \sigma\epsilon(T_H + T_S)(T_H + T_S)^2 F_{H->S} A \tag{S9}$$

Where σ is the Stefan's constant with value of 5.67×10^{-8} $\mathrm{Wm^{-2}K^{-4}}$, ϵ is the emissivity, T_H and T_S are the temperature of the heater and sensor sides. Further, $F_{H->S}$ is the view factor and A is the area of the membranes facing each other. The emissivity is assumed to be one. The view factor $F_{H->S}$ for the membrane area of (30×0.2) μm^2 can be calculated as described in the reference.[3] Considering our device's characteristics, we get $F_{H->S}A \approx 8.71 \times 10^{-12}$ μm^2. Therefore, at the highest bias $\Delta T_H = 200$ K and $\Delta T_S = 20$ K, G_{rad} is 1.28×10^{-10} W/K, which is below the measurement sensitivity of the thermal bridge (8×10^{-10} W/K).

Temperature fluctuations: We have a precision of 0.01 K with the stage temperature controller, however, there can be some temperature fluctuations in the sample. To avoid this, a stabilization time of 1 hour was usually taken for each temperature measurement.

Temperature coefficient of resistance (α): One of the largest sources of error in our case is the measurement of temperature coefficient of resistance ($\alpha = \frac{1}{R}\frac{dR}{dT}$). From the fit of $\frac{dR}{dT}$, there is an error of approximately 10% arising from the noise in the resistance measurement. The noise in the resistance measurement comes from the standard deviation of the 200 data points taken over 1 second at a negligible current to avoid heating (100 nA) using a lock-in amplifier. This noise in the lock -in amplifier – AC output voltage can be further estimated as NER/R_S, where NER is the noise equivalent resistance.[2] Since resistance is calculated using the voltage measured by the lock-in amplifier, therefore, NER/R_S can be calculated

as the noise in the voltage $\delta V/V$ with δV being the fluctuations in the measured voltage. At 300 K, with an applied potential of 0.2 mV, the noise level (δV) is measured to be 1.5 $\times$ 10^{-7} corresponding to $\delta V/V$ of 5 $\times$ 10^{-4}. This error in resistance contributes twice in the measurement of nanowire thermal conductance G_n. Once, towards the measurement of resistance as a function of temperature R(T) for TCR and second, towards the measurement of resistance as a function of dissipated power R(P) while applying thermal biases.

Application of thermal bias: While applying thermal biases, the temperatures measured on the heater T_H and sensor sides T_S with the combined errors of resistance measurement and temperature coefficient of resistance result in an error percentage of 8%.

Error in asymmetry: The fabrication disparities in the two bridges of the device lead to different beam conductance of the two bridges. This asymmetry factor induces an approximate error of 5 to 6% in the thermal conductance measurement.

Final error in thermal conductance: The final error in the measured thermal conductance can be accounted by propagating all the errors mentioned above in this equation:

$$\Delta G_n = \sqrt{\left\{G_B \frac{B}{(B-A)^2}\Delta B\right\}^2 + \left\{G_B \frac{B}{(B-A)^2}\Delta A\right\}^2 + \left\{\frac{G_n}{G_B}\Delta G_B^2\right\}} \tag{S10}$$

Where A = dT_H/dP, B= dT_S/dP are the slopes of heater and sensor side, G_B is the beam conductance, and G_n is the nanowire thermal conductance. The systematic errors of the experimental setup from different sources reported above propagate leading to about 13 – 15% error in our thermal conductance measurements.

Measurement sensitivity of the experimental setup

Since the limit of the thermal sensitivity of the thermal bridge method is 8 $\times$ 10^{-10} W/K, our setup is sensitive to the thermal conductance values above this. However, for detecting thermal rectification, our experimental setup should be sensitive to the difference in the thermal conductance in forward and reverse bias conditions. To calculate the smallest difference

in the thermal conductance that is detectable by our experimental setup, we can estimate the noise-equivalent thermal conductance, NEG_n of our setup. This should correspond to the error of the temperature rise that can be measured using our setup on the sensor side R_s at different bias conditions. Estimation of noise-equivalent thermal conductance for different applied thermal biases is given by Shi *et al.*[2] as:

$$NEG_n = G_b \frac{NET}{\Delta T_H - \Delta T_S} \tag{S11}$$

Where NET is the noise-equivalent temperature rise, G_B is the beam thermal conductance, ΔT_H and ΔT_S are the temperatures rise on the heater and sensor sides. To estimate NEG_n, we take NET as the error of the temperature rise on the sensor side at different thermal biases. Putting the values of G_b at different temperature biases, we get a NEG_n of 1.33×10^{-10} W/K at electrical $\Delta T = 80$ K and of 1.31×10^{-10} W/K at ΔT of 200 K. Therefore, this estimated NEG_n corresponds to the smallest detectable difference in thermal conductance measurable by our experimental setup.

Temperature dependent thermal conductivity of a 300 nm thick ZB GaAs nanowire

We could measure only the temperature dependence of the GaAs ZB segment with bigger diameter (see Fig. S3 below). In this case, we have measured the thermal conductivity in a telescopic NW which had a broken thin part (optical image in the inset of Fig. S3).

Rectification factor of NW_1 and NW_2 300K - 400 K , $\Delta T = 50$K

Using the definition of rectification factor given in Eq. 3 in the main manuscript, rectification values are calculated for the three measured telescopic nanowires. For NW_1 and NW_2, rectification values were calculated as a function of base temperature in the range 300 to 340 K at an applied bias of 50 K, based on the thermal conductance curve shown in Fig. 2D in the

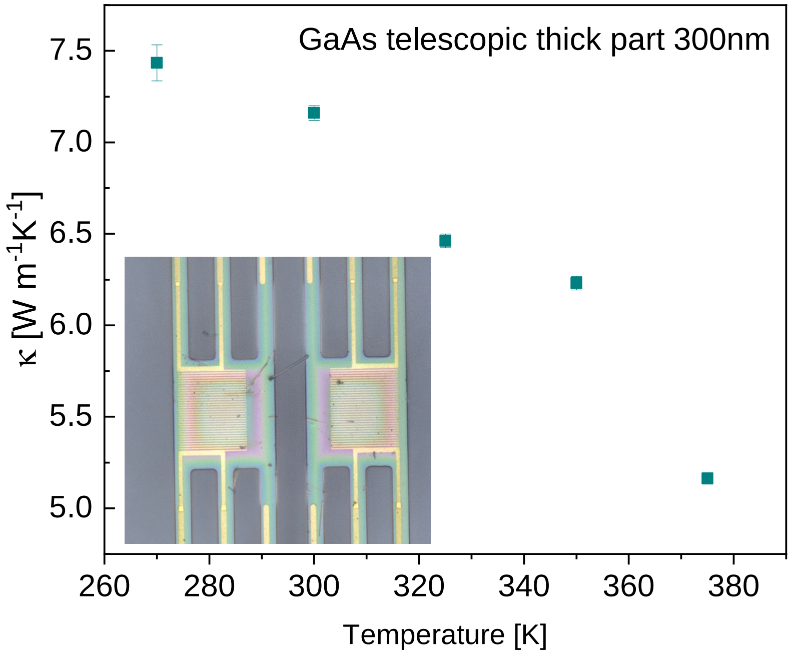

Figure S3. Measured trend of thermal conductivity of a thick ZB GaAs NW as a function of temperature. Inset: Optical image of the measured ZB GaAs NW

main manuscript. As can be seen in Fig. S4, the rectification values for NW_1 in red triangles are scattered between small negative values to 25%. It must be noted that four out of six data points exhibit positive rectification. Although this does not describe any trend, there is still an indication of rectification, which is indeed confirmed by measuring NW_2. Rectification values of the order of 10% were obtained consistently within the temperature range except for 380 K. For NW_1, the large fluctuation might arise from the fact that the device's asymmetry is not considered for rectification calculations. This was the first system to be measured, and the measurement protocol was not fully established for rectification measurements till then. Subsequent measurements were taken considering the asymmetry factor and averaging the measurements in the lock-in on the sensor side for 1 s (0.5 s before) to correct for the device's response time being 0.5 s. We inculcated these improved measurement conditions for experiments on NWs 2 and 3.

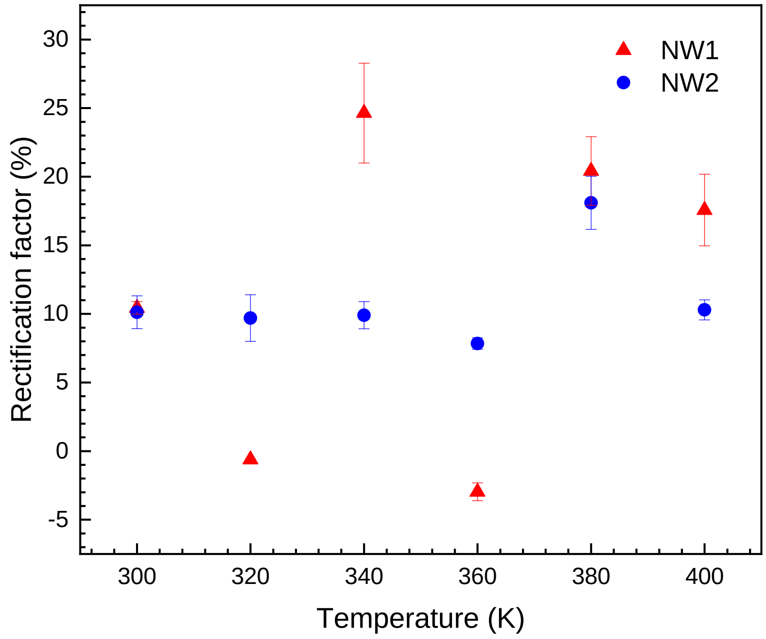

Figure S4. Thermal rectification factors of telescopic nanowires 1 and 2 as a function of base temperatures from 300 to 400 K at an applied bias of 50 K.

Computational methods

We performed density functional theory (DFT) calculations with the VASP code[4] and projector augmented waves[5,6] and the local density approximation (LDA). We computed interatomic force constants (IFC) by finite differences with the phonopy[7] and the third order.py[8] code. We used a $5 \times 5 \times 5$ ($4 \times 4 \times 4$) supercell for the second-order IFCs of the zinc blende (wurtzite) phase and a ($4 \times 4 \times 4$) supercell for the third-order IFCs of both polytypes. We then used the ShengBTE code[8] to solve the Boltzmann transport equation (BTE) beyond the Relaxation Time Approximation (RTA) and accounting self-consistently for the boundary scattering resulting from the radial confinement imposed by the NW geometry. See Ref.[9] for full details of these calculations.

The thermal conductivity as a function of the temperature of a zinc blende and a wurtzite GaAs NW with a diameter of 300 and 90 nm, respectively, were used as input data to

solve numerically the one-dimensional heat equation with a finite-element method.[10] When present in the simulations, the thermal boundary resistance (TBR) was accounted for within the diffuse mismatch model (DMM).[11]

Exploration of the length-radius phase-space

In order to test the robustness of our conclusions, we have carried out an additional set of simulations where we varied the length, L, and the diameter, Φ, of the two NW segments. In this way, while we still rely on the computed ab initio bulk $\kappa(T)$, we have different conductances, which are the magnitudes that ultimately matter when it comes to determining the thermal rectification, γ. We first varied the segments' lengths, L_{ZB} and L_{WZ}, taking the nominal value of the diameters $\Phi_{ZB} = 300$ nm and $\Phi_{WZ} = 90$ nm; next we varied the segments' diameters, taking the nominal values of the lengths $L_{ZB} = 2.9\mu$m and $L_{WZ} = 5.8\mu$m. These simulations were performed both with and without the TBR, as computed within the DMM (see main text).

The results are displayed in Figure S5. As it can be seen there, whenever the TBR is not included, we obtain values of γ that are lower than 1% and that in some case are even negative (i.e., predicting rectifications opposite to the ones observed experimentally). Conversely, when the TBR is accounted for, even if we depart significantly from the nominal values of L and Φ, and thus of the segments' conductances, we obtain values of γ that are of the same order of those observed experimentally. Therefore, whenever we considered physically sound $\kappa(T)$ and TBR values (and not necessarily only those directly resulting from our ab initio calculations), we always found that the order of magnitude of the measured thermal rectification can only be recovered when the effect of the TBR is considered. This suggest that our conclusions on the role of the TBR seems to be a rather general fact that does not rely on fine details of the modelling.

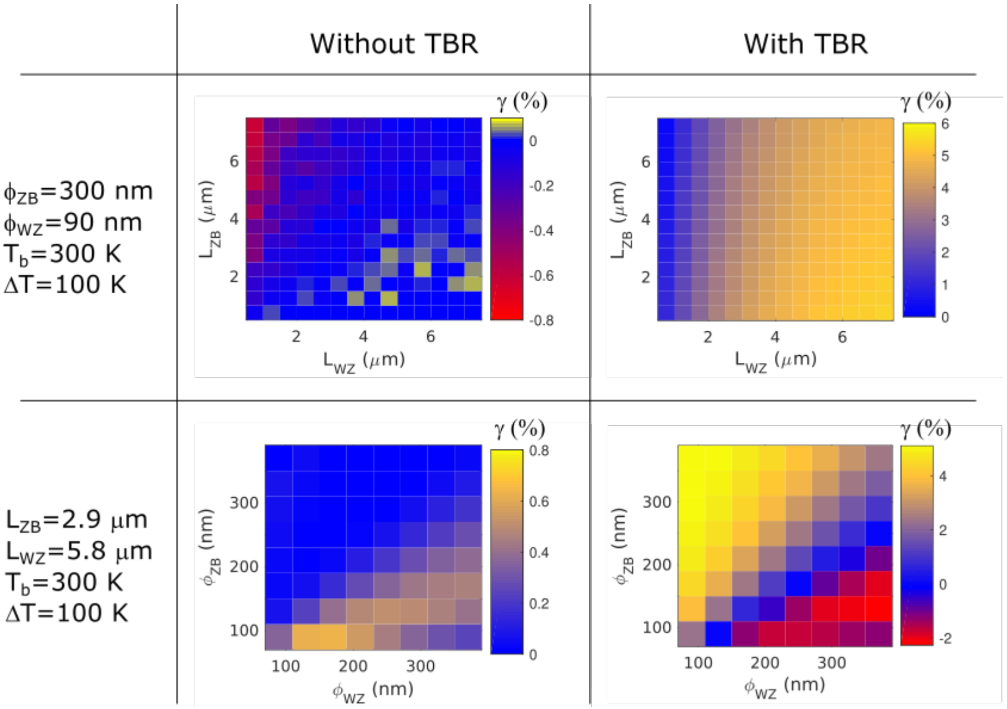

Figure S5. Thermal rectification, γ, at fixed $T_{base} = 300$ K and $\Delta T = 100$ K, (left) without and (right) with TBR computed from the DMM. Top row: γ as a function of the segments' lengths, L_{ZB} and L_{WZ}, taking the nominal value of the diameters, $\Phi_{ZB} = 300$ nm and $\Phi_{WZ} = 90$ nm. Bottom row: γ as a function of the segments' diameters, Φ_{ZB} and Φ_{WZ}, taking the nominal value of the lengths, $L_{ZB} = 2.9\mu$m and ${}_{LWZ} = 5.8\mu$m.

Thermal equivalent circuit of a telescopic nanowire suspended on a thermal bridge device

For the experimental system, a thermal circuit comprising conductances with the corresponding heat currents is introduced, assuming the background conduction is negligible. The equivalent thermal circuit diagram is shown in Fig. S7 for our experimental system of the telescopic nanowire in the forward configuration, i.e. heat flowing from the thick to the thin part. $\mathrm{T}_{\mathrm{H,\,Elec}}$ and $\mathrm{T}_{\mathrm{S,\,Elec}}$ are the temperatures measured at the platforms on the heater and sensor side through electrical measurements. $\mathrm{T}_{\mathrm{H,\,Raman}}$ and $\mathrm{T}_{\mathrm{S,\,Raman}}$ are the temperatures measured on the hot and cold sides by the Raman laser probe while electrically applying thermal bias. P_{H} is the power dissipated on the heater side. T_{b} is the base temperature of the system. $P_{\mathrm{b,\,H}}$ and $P_{\mathrm{b,\,S}}$ are power dissipated in the beams on the heater

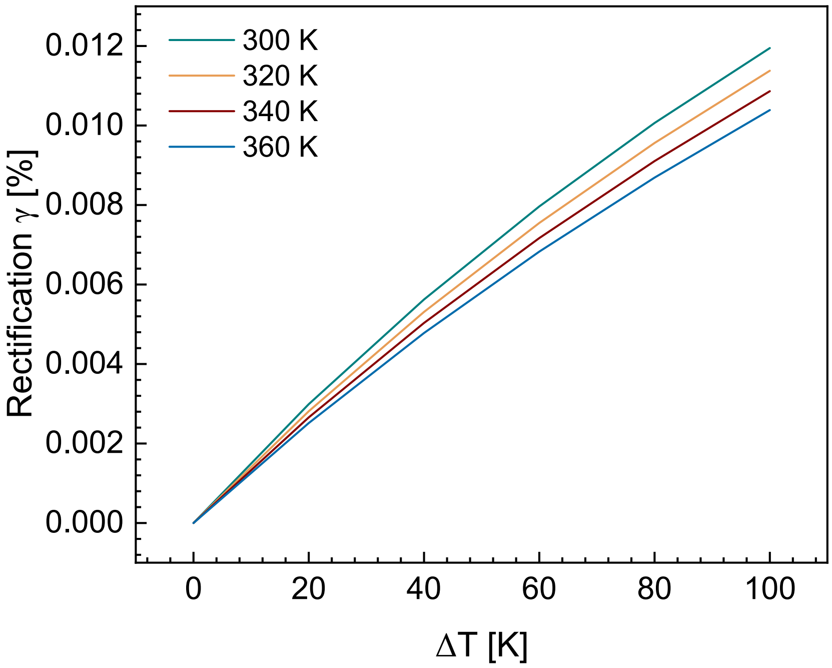

Figure S6. Rectification factors obtained applying the model of Dames,[12] i.e. neglecting the TBR and taking the temperature dependence of the thermal conductivity, $\kappa(T)$, of the two segments to be exponential (we obtained $n_1 = -0.6593$ and $n_2 = -0.7549$, following the nomenclature used in the main text). These data should be compared with those of Fig. 4a in the main text, where TBR is also neglected, but the heat equation is solved numerically without assuming any functional dependence for $\kappa(T)$.

and sensor sides, respectively. $G_{\text{c, H}}$ and $G_{\text{c, S}}$ are contact conductances on the heater and sensor sides. G_{NW} is the nanowire conductance comprising the total conductance of the thick and thin parts of the telescopic nanowire. $G_{\text{b, H}}$ and $G_{\text{b, S}}$ are the beam conductances on the heater and sensor side, respectively.

To estimate the effect of thermal contact resistance, Raman thermometry measurements were carried out at points of contact 1 and 2 between the wire and the device membranes and the temperature profiles were plotted as reported in Fig. 5 of the main manuscript.

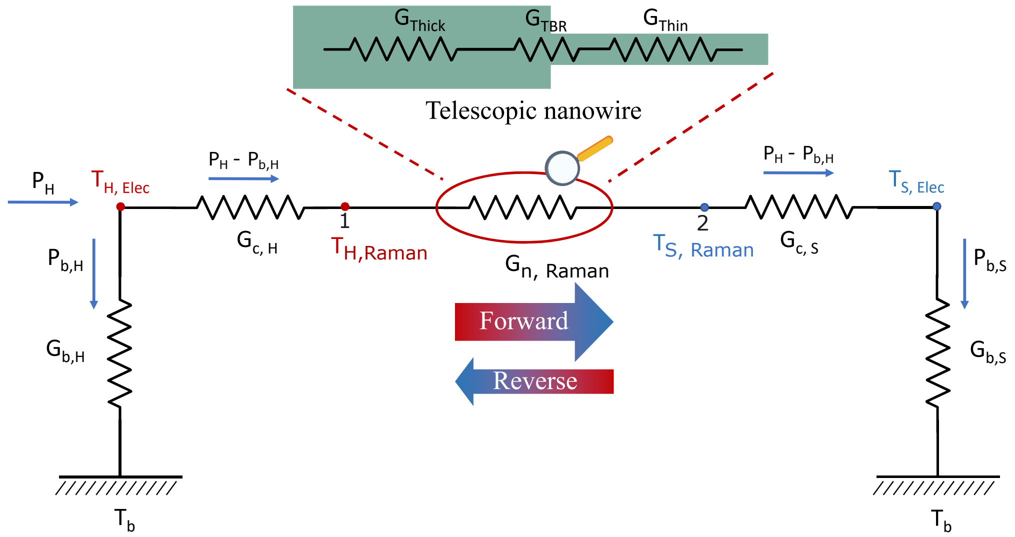

Figure S7. Thermal equivalent circuit model for a telescopic nanowire suspended on a thermal bridge device showing the powers dissipated in constituting parts of our thermal circuit with corresponding temperatures as well as marking the forward and reverse bias conditions.

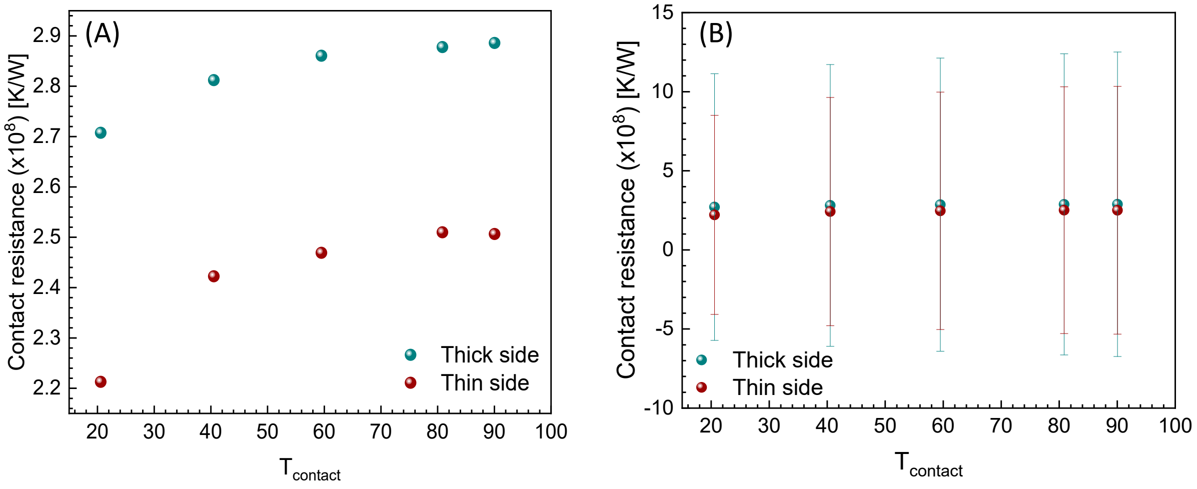

Figure S8. Thermal contact resistance measured at the contact between the thick/thin parts of the telescopic NW and the SiN_x membranes through Raman thermometry on a thermal bridge (A) with error bars (B) without error bars.

References

(1) Kim, W.; Dubrovskii, V. G.; Vukajlovic-Plestina, J.; Tütüncüoglu, G.; Francaviglia, L.; Güniat, L.; Potts, H.; Friedl, M.; Leran, J. B.; Fontcuberta I Morral, A. Bistability of Contact Angle and Its Role in Achieving Quantum-Thin Self-Assisted GaAs nanowires. *Nano Lett.* **2018**, *18*, 49–57.

(2) Shi, L.; Li, D.; Yu, C.; Jang, W.; Kim, D.; Yao, Z.; Kim, P.; Majumdar, A. Measuring thermal and thermoelectric properties of one-dimensional nanostructures using a microfabricated device. *J. Heat Transfer* **2003**, *125*, 881–888.

(3) Howell, J. R.; Mengüç, M. P.; Daun, K.; Siegel, R. *Thermal radiation heat transfer*; CRC press, 2020.

(4) Kresse, G.; Furthmüller, J. Efficient iterative schemes for ab initio total-energy calculations using a plane-wave basis set. *Phys. Rev. B* **1996**, *54*, 11169–11186.

(5) Blöchl, P. E. Projector augmented-wave method. *Phys. Rev. B* **1994**, *50*, 17953–17979.

(6) Kresse, G.; Joubert, D. From ultrasoft pseudopotentials to the projector augmented-wave method. *Phys. Rev. B* **1999**, *59*, 1758–1775.

(7) Togo, A.; Chaput, L.; Tanaka, I. Distributions of phonon lifetimes in Brillouin zones. *Phys. Rev. B* **2015**, *91*, 094306.

(8) Li, W.; Carrete, J.; Katcho, N. A.; Mingo, N. ShengBTE: a solver of the Boltzmann transport equation for phonons. *Comp. Phys. Commun.* **2014**, *185*, 1747–1758.

(9) Raya-Moreno, M.; Rurali, R.; Cartoixà, X. Thermal conductivity for III-V and II-VI semiconductor wurtzite and zinc-blende polytypes: The role of anharmonicity and phase space. *Physical Review Materials* **2019**, *3*, 084607.

(10) Cartoixà, X.; Colombo, L.; Rurali, R. Thermal Rectification by Design in Telescopic Si Nanowires. *Nano Lett.* **2015**, *15*, 8255–8259.

(11) Swartz, E. T.; Pohl, R. O. Thermal boundary resistance. *Reviews of modern physics* **1989**, *61*, 605.

(12) Dames, C. Solid-state thermal rectification with existing bulk materials. *Journal of Heat Transfer* **2009**, *131*.
